# Supplementary material for: The Systems Biology Research Tool: evolvable open-source software
Source: BMC Syst Biol. 2008 Jun 29;2:55. doi: 10.1186/1752-0509-2-55 (PMC2446383; doi:10.1186/1752-0509-2-55)
Supplement: Additional file 1 — SBRT Archive. An archive of the current version of the Systems Biology Research Tool. [file 1752-0509-2-55-S1.zip › sbrt-1.4.0/doc/developers_guide/api/index-files/index-2.html]

B-Index


|  |  |  |
| --- | --- | --- |
| **Overview** \| Package \| Class \| Use \| **Tree** \| **Deprecated** \| **Index** \| **Help** | |  |
| **PREV LETTER**   **NEXT LETTER** | **FRAMES**    **NO FRAMES**     **All Classes** |  |


A B C D E F G H I J K L M N O P Q R S T U V W X Y Z 

---


## **B**

**BACK\_SLASH** - Static variable in class sbrt.shell.text.SpecialChars: \ **BiggBracketParserV1** - Class in sbrt.shell.text.fba: This class is used to parse bracketed expressions contained in SBML files from the BiGG Database. **BiggBracketParserV1()** - Constructor for class sbrt.shell.text.fba.BiggBracketParserV1: **BiggRxnNodeParser** - Class in sbrt.shell.text.fba: This class is used to make a direct translation from SBML 'reaction' nodes into Systems Biology Research Tool objects. **BiggRxnNodeParser()** - Constructor for class sbrt.shell.text.fba.BiggRxnNodeParser: Constructs a new SBML reaction node parser. **BiggSbmlReader** - Class in sbrt.shell.proc.fba: This class is used to read SBML files downloaded directly from the BiGG Database. **BiggSbmlReader(String)** - Constructor for class sbrt.shell.proc.fba.BiggSbmlReader: Constructs a new SBML file reader. **BiggSbmlReaderManager** - Class in sbrt.shell.mng.fba: This class is used to manage the reading of SBML files obtained from the BiGG Database. **BiggSbmlReaderManager()** - Constructor for class sbrt.shell.mng.fba.BiggSbmlReaderManager: **BiggSbmlTranslater** - Class in sbrt.shell.proc.fba: This class is used to translate SBML files from the BiGG Database into the Systems Biology Research Tool's *FBA Reaction Files* and *FBA Catalyst Files*. **BiggSbmlTranslater()** - Constructor for class sbrt.shell.proc.fba.BiggSbmlTranslater: Constructs a new SBML translater. **BiggSbmlTranslaterManager** - Class in sbrt.shell.mng.fba: This class is used to manage the translation of SBML files from the BiGG Database into the Systems Biology Research Tool's *FBA Reaction Files* and *FBA Catalyst Files*. **BiggSbmlTranslaterManager()** - Constructor for class sbrt.shell.mng.fba.BiggSbmlTranslaterManager: **BooleanFormat** - Interface in sbrt.shell.text: This interface is used to represent a format for boolean values. **BooleanRelation**<E> - Class in sbrt.kernel.util: **BooleanRelation(Collection<E>)** - Constructor for class sbrt.kernel.util.BooleanRelation: **BoolRelFormatV1**<E> - Class in sbrt.shell.text: This interface is used to represent boolean relationship formats. **BoolRelFormatV1(SimpleFormat<E>)** - Constructor for class sbrt.shell.text.BoolRelFormatV1: Constructs a new boolean relation format object that uses the provided format for its elements. **BoundedIntervalFormatV1** - Class in sbrt.shell.text.math: This class is a concrete implementation of `IntervalFormat`. **BoundedIntervalFormatV1()** - Constructor for class sbrt.shell.text.math.BoundedIntervalFormatV1: Constructs a new interval format. **breakPoint()** - Method in class sbrt.kernel.io.AbstractResultsManager: Does nothing. **breakPoint()** - Method in interface sbrt.kernel.io.ResultsManager: Signals to this manager that a point of discontinuity or change has been reached. **breakPoint()** - Method in class sbrt.shell.io.AbstractMapOutputFile: Writes a line of `#` symbols to this file. **breakPoint()** - Method in class sbrt.shell.io.fba.ColumnOrFbaOptOutputFile: Writes a line of `#` symbols to this file. **breakPoint()** - Method in class sbrt.shell.io.fba.RowOrFbaOptOutputFile: Writes a line of `#` symbols to this file. **breakPoint()** - Method in class sbrt.shell.io.fba.SerFbaOptOutputFile: Does nothing. **breakPoint()** - Method in class sbrt.shell.io.MultiOutputFiles: Closes the current file if one exists. **breakPoint()** - Method in class sbrt.shell.io.SerializedOutputFile: Does nothing. **breakPoint()** - Method in class sbrt.shell.io.TextOutputFile: Writes a line of `#` symbols to this file.

---


|  |  |  |
| --- | --- | --- |
| **Overview** \| Package \| Class \| Use \| **Tree** \| **Deprecated** \| **Index** \| **Help** | |  |
| **PREV LETTER**   **NEXT LETTER** | **FRAMES**    **NO FRAMES**     **All Classes** |  |


A B C D E F G H I J K L M N O P Q R S T U V W X Y Z 

---
